# Close of the Volume

**Published:** 1856-08

**Authors:** 


### EDITORIAL AND MISCELLANEOUS.

### CLOSE OF THE VOLUME.

With this number, our Journal closes the first year of its existence, at which time we conceive it not to be inappropriate to say something of ourselves.

Whether we have succeeded in doing any thing for the advancement of medical science, it is not for us to say, but we think it not presumptuous to conclude that our promise to establish a respectable Medical Journal has been fulfilled, and upon the whole we have attained a position by no means discouraging.

Our confidence in those to whom we looked for original contributions, has not been misplaced, and up to the present time, we have had a steadily increasing list of contributors and subscribers, and we have an abiding reliance upon the profession, for their liberal aid in sustaining us for the future, promising upon our part, if not talent, at least energy, fairness and independence.

Owing to the legislation of Georgia, in reference to medical matters, and the public sentiment manufactured in many cases, and pandered to in others, by penny politicians, the science of medicine proper, is placed here under great disabilities, and if united action and organized effort is required any where, it does seem to us to be most urgently demanded of the medical profession of this State, and, indeed, when we think of the amount of medical talent, learning, professional and personal influence, scattered throughout the State of Georgia, we can come to no other conclusion, than that we remain in our present position *solely* from an inexcusable lethargy and want of organization and concentration of the power and influence which properly belong to us, the united exertion of which would soon effect a *reform* in the position of medicine, worthy of her exalted merits and large claims, not only upon the justice, but the generosity of the public.

*Regular* medical men, those who are devoted to the honorable and conscientious pursuit of a noble profession—based upon the accumulations of science, as the result of her investigations into every department of nature's domain, for thousands upon thousands of years—are usually gentlemen with more than an ordinary amount of intelligence and influence, and this is *especially* the case in the country; (for our doctrine is, that the mass of men are better in the country; and particularly do we find less selfishness and less professional jealousy, the existence of which, exerts so baneful an influence, but which may be looked upon as the legitimate result of the fierce competition which we so often unfortunately witness in towns where medical men "most do congregate;") and it is then, to the latter that we have to look to a great extent, for the exertion of that kind of influence which will *compel* our legislators, individually and collectively, to respect and acknowledge the just demand of the regular profession to be considered as the *only* representatives of the great science of healing—in which every thing is embraced which can furnish aid in the relief of disease, or suggest means of its *prevention*, or for the prolongation of human life—in contradistinction to the false claims of the ignorant or unprincipled pretenders to *special systems*, that bear about the same relation to the *science of medicine* that Mormonism and spiritualism sustain to *true* religion.

In what we have said with regard to the respective influence exerted by town and country practitioners, we would not be understood as casting a slur upon the former, the rule to which we have alluded being applicable to those of every profession and calling — so far as *general public sentiment* is concerned, a lessening of influence in proportion to their congregation—when more diffused, each having his circle of influence enlarged, and then by *united action*, a more extensive and efficient power is exerted over larger sections and masses.

We earnestly hope that the medical men of Georgia, and indeed of the whole South, will soon arouse to a consciousness of their power, and a proper sense of the importance of the exertion of that power, and especially that the great mass of the profession with whom rests the influence which could give us all the advantages we ask, will not trust to the efforts of the comparatively few who are found in the larger towns, who, though under circumstances more favorable for the organization of societies, &c., are less apt to act in concert, and really possess a smaller proportional efficiency in controling legislative action.

One of the prominent objects in this enterprise has been to advance the interests of the profession; for this we have labored, and are willing to spend and be spent; and while we feel grateful for the assistance and indulgence so liberally extended to us heretofore, in the attempt to discharge the arduous and responsible duties of the editorial chair, with promises of renewed energy upon our part, we must again urgently solicit large aid and a generous judgment upon the part of those who have a common interest with us, in the advancement of science and its proper appreciation upon the part of the world. The success of our Journal is (we can now say, without presumption or apprehension) one of the permanent facts in the history of Southern medicine, having an equally solid foundation, with the successful medical Institution, whose claims it incidentally advocates.

---

### TO SUBSCRIBERS.

Notwithstanding the determination of the projectors of this Journal to continue it, under any circumstances, and the fact that it was not undertaken with any expectation of deriving pecuniary profit from its publication, it is still expected that those who receive it, will not allow us to do more than give our labor gratuitously: this, as before intimated, we are willing, and expect, to do.

For the remuneration of the publishers, to whom we are re sponsible, (they having no direct interest in the work,) we look to our subscription list; and we learn from those who manage the financial department, that there is no difficulty in meeting the monthly payments to the publishing office, provided our regular subscribers comply with the terms upon which the Journal is issued, and sent to them.

While many have paid, *a large number are in arrears*, and we hope that the bare announcement of this fact will prevent the necessity of again recurring to this subject.

